# Dynamic response of polar nanoregions under an electric field in a paraelectric KTa_0.61_Nb_0.39_O_3_ single crystal near the para-ferroelectric phase boundary

**DOI:** 10.1038/srep13751

**Published:** 2015-09-03

**Authors:** Hao Tian, Bo Yao, Lei Wang, Peng Tan, Xiangda Meng, Guang Shi, Zhongxiang Zhou

**Affiliations:** 1 Department of Physics, Harbin Institute of Technology, Harbin 150001, China

## Abstract

The dynamic response of polar nanoregions under an AC electric field was investigated by measuring the frequency dependence of the quadratic electro-optic (QEO) effect in a paraelectric KTa_0.61_Nb_0.39_O_3_ single crystal near the para-ferroelectric phase boundary (0 °C < *T*-*T*_*c*_ < 13 °C). The QEO coefficient *R*_11_ − *R*_12_ reached values as large as 5.96 × 10^−15^ m^2^/V^2^ at low frequency (500 Hz) and gradually decreased to a nearly stable value as the frequency increased to 300 kHz. Furthermore, a distortion of the QEO effect was observed at low frequency and gradually disappeared as *R*_11_ − *R*_12_ tended towards stability. The giant QEO effect in the KTa_0.61_Nb_0.39_O_3_ crystal was attributed to the dynamic rearrangement of polar nanoregions and its anomalous distortion can be explained by considering the asymmetric distribution of polar nanoregions.

Recently, KTa_1−*x*_Nb_*x*_O_3_ (KTN)-based single crystals have attracted much scientific attention owing to their excellent quadratic electro-optic (QEO) and electrostrictive effects^1^,^2^,^3^,^4^,^5^,^6^,^7^,^8^,^9^. Abundant devices such as electro-optic (EO) switches, deflectors and modulators based on KTN crystals have been designed and fabricated^4^,^6^,^7^,^8^,^9^. Based on the large QEO effect, a voltage-controlled diffraction efficiency as high as 79% can be achieved in Mn: KTN or Fe: Mn: KTN^10^,^11^. Besides, the Li: KTN crystal has been used to develop scale-free optics, which could lead to a completely new paradigm for ultraresolved imaging and microscopy^12^,^13^. The origin of the giant QEO effect and novel scale-free optics in paraelectric KTN have been attributed to the presence of polar nanoregions (PNRs) near the para-ferroelectric phase boundary^1^,^2^,^3^,^12^,^13^. Unfortunately, the mechanism with which PNRs contribute to these phenomena is still unclear. Present research on the QEO effect in KTN mainly investigates DC or low-frequency (<500 Hz) electric fields^1^,^2^,^3^,^4^, whereas research on the QEO effect under higher-frequency electric fields should be performed to promote the implementation of KTN in broadband optical communications. Furthermore, the investigation of the QEO effect under wide-frequency-range electric fields could facilitate the interpretation of the dynamic response of PNRs, which is also associated with other related interesting phenomena, like scale-free optics.

The KTN crystal is the solid solution of KTaO_3_ and KNbO_3_. It can exist in the cubic, tetragonal or orthogonal phase at room temperature, depending on the composition fraction *x*. Owing to the compositional disorder of the Ta and Nb ions in the B-sites, spontaneous polarization mainly induced by the displacement of the Nb ions arises in local regions above the Curie point *T*_*c*_. The average size of these local regions is in the nanoscale, hence they are usually called polar nanoregions (PNRs). As widely reported, PNRs have been observed in paraelectric KTN crystals near the para-ferroelectric phase boundary^14^,^15^. When the temperature decreases to the Burns temperature *T*_*B*_, the dipole moments induced by the B site off-centered Nb ions become correlated. The PNRs represent dynamic polarization fluctuation with a finite lifetime shorter than the average acoustic phonon period, so they could not affect the dielectric property of the crystal at the low frequency. As the temperature further decreases to an intermediate temperature T^*^, the strengthened correlations increase the lifetime of PNRs, eventually stabilizing the dynamic PNRs into “static” PNRs (with permanent polarization fluctuation). The reorientation of the “static” PNRs gives rise to the relaxor behavior that starts at T^*^, which could be characterized by the deviation of the real part of the dielectric constant from a Curie-Weiss law^15^. Accordingly, at the temperature range *T*_*c*_ < *T* < T^*^, the PNRs start to affect the QEO and electrostrictive effects of the crystal. Finally, when the temperature decreases to *T*_*c*_, the PNRs transform into large ferroelectric domains, indicating the transition into the tetragonal phase.

In this study, we investigated the dynamic response of PNRs to an electric field by measuring the frequency dependence (500 Hz−300 kHz) of the QEO effect in a KTa_0.61_Nb_0.39_O_3_ crystal at the temperature range 0 °C < *T*-*T*_*c*_ < 13 °C (*T*_*c*_ + 13 °C < T^*^). The contribution of the PNRs to the QEO effect was estimated quantitatively. Additionally, a distortion of the QEO effect was observed at low frequency and gradually disappeared as the frequency increased. A phenomenological model is proposed to understand the mechanism with which PNRs contribute to the QEO effect.

## Experimental

The KTN crystal was grown with an improved top-seeded solution growth method^16^. The sample was cut into a cuboid of dimensions 3.30 × 2.20 × 0.98 mm^3^ along the crystallographic [001], [010] and [100] directions respectively. The 3.30 × 2.20 mm^2^ faces were polished and silver paint was coated on the 3.30 × 0.98 mm^2^ faces to form electrodes. The refractive indices of the samples were measured by a spectroscopic ellipsometer. The temperature dependence of the dielectric constant was measured with an LCR meter (E4980A, Agilent Technologies, USA) to determine *T*_*c*_. The evolution of the PNRs with the temperature change was observed with a polarizing microscope (Axioskop 40, Zeiss, Germany).

**Figure 1:**
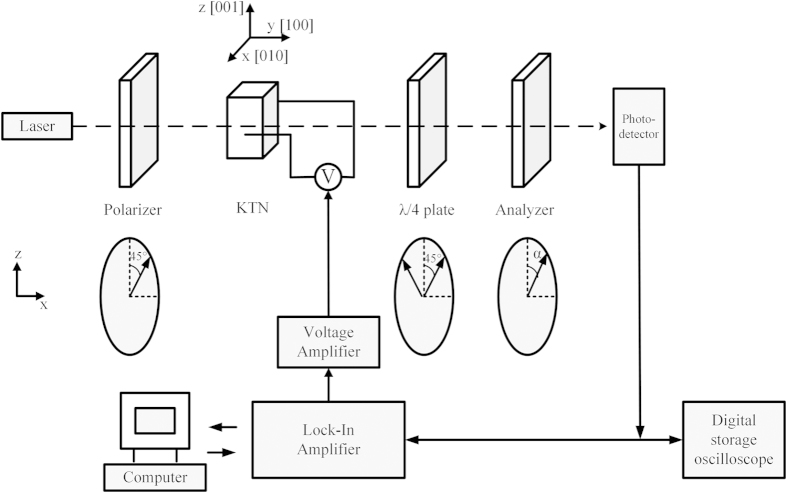
The schematic of Senarmont compensator method. The light transmitted along the crystallographic [100] (0.98 mm) direction and the electric field was applied along the [010] (2.20 mm) direction. The electric field and the light intensity after the Analyzer were monitored by the oscilloscope.

The QEO coefficients *R*_11_ − *R*_12_ at the wavelength 632.8 nm were measured by the Senarmont compensator method^17^. The components were arranged as the schematic shown in Fig. 1. The output AC voltage signal 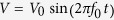 of a frequency *f*_0_ from the lock-in amplifier (SR830 and SR844, Stanford Research Systems, USA) was amplified by the voltage amplifier (WMA-300, Falco, Netherlands), then applied to the crystal along the crystallographic [010] (2.20 mm) direction. The light intensity after the Analyzer was transformed to electric signal by the photo-detector (HCA-S-400 M-SI, Femto, Germany), then filtered by the lock-in amplifier to obtain the QEO signal. As the angle *α* changed from 0 to π, the light intensity after the Analyzer gradually changed in the range from *I*_max_ to *I*_min_. Before the measurement, the angle *α* was set to certain angle to make the light intensity after the Analyzer equal (*I*_max_ − *I*_min_)/2. Then the electric field was applied to induce birefringence between the *x* and *z* direction. The phase change ΔΦ induced by this birefringence led to the light intensity variation Δ*I*. The QEO coefficient *R*_11_ − *R*_12_ could be obtained by equation (1).


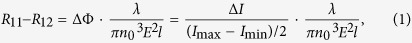


where *λ* is the wavelength and *l* is the length of the crystal along the [100] direction, *n*_0_ is the refractive index of the crystal without the electric field *E*. Besides, the electric field and the light intensity after the Analyzer were monitored by the oscilloscope. More details about the Senarmont compensator method are discussed in Ref. 17.

## Results and Discussions

**Figure 2:**
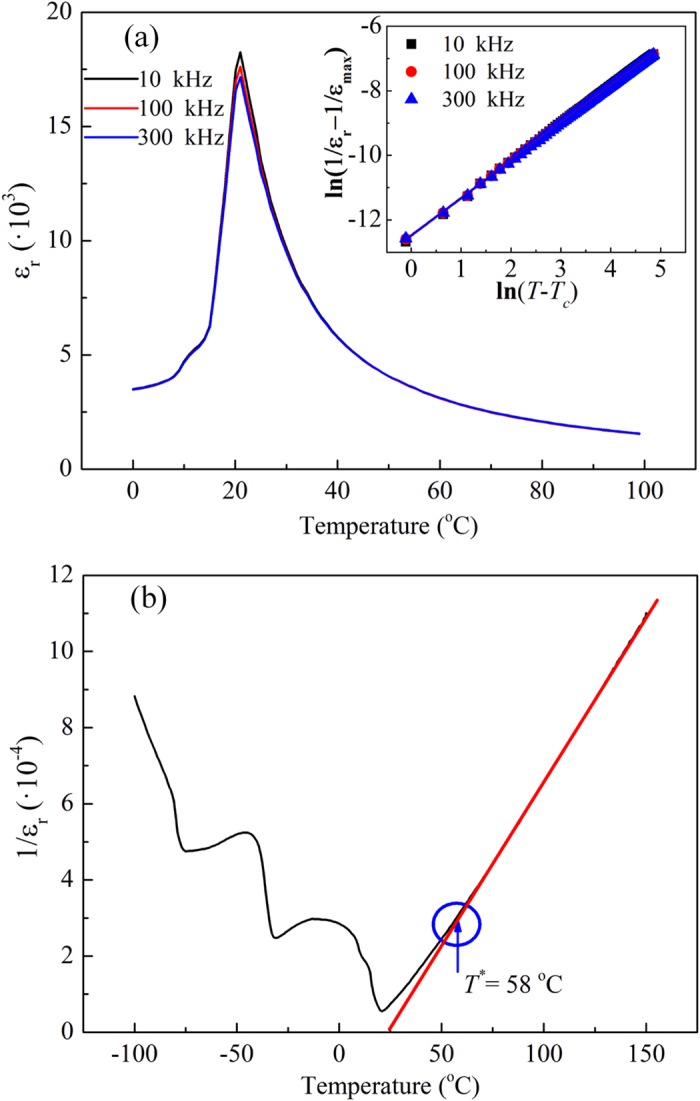
(**a**) The temperature dependence of the dielectric constant *ε*_*r*_ of the KTa_0.61_Nb_0.39_O_3_ crystal at 10 kHz, 100 kHz and 300 kHz. Fitting results of equation (2) to determine the relaxation factor *γ* are shown in the inset. (**b**) The black curve displays the temperature dependence of the reciprocal of the dielectric constant *ε*_*r*_. The red curve is the fitting result of the Curie-Weiss law for *ε*_*r*_ at high temperatures above the intermediate temperature T^*^, which is indicated by the blue arrow.

The temperature dependence of the dielectric constant *ε*_*r*_ of the crystal at 10 kHz, 100 kHz and 300 kHz is shown in Fig. 2(a). The peaks of the curves indicate that the *T*_*c*_ of the sample was 21.0 °C and its composition was KTa_0.61_Nb_0.39_O_3_, which was determined by the empirical formula presented in Ref. 18. The maximum dielectric constant was 18000, which confirmed the high quality of the crystal. The sharp peaks appear in the same position for all the examined frequencies, which is why the para-ferroelectric phase transition of the KTN crystal has been regarded as non-relaxation in the past decades. However, the relaxation factor *γ*, defined by equation (2)^19^,^20^, was found to be approximately 1.16, according to the fitting results shown in the inset of Fig. 2(a).


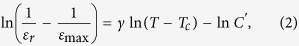


where *C´* is the modified Curie-Weiss constant and *ε*_max_ is the maximum dielectric constant. According to R. Clarke and J. C. Burfoot^20^, 1 < *γ* < 2 indicates that the para-ferroelectric phase transition of KTN actually corresponds to a slight relaxation, which implies the existence of PNRs above *T*_*c*_. To determine the temperature range *T*_*c*_ *<* *T* *<* T^*^, at which the PNRs contribute to the QEO effect, the intermediate temperature T^*^ = 58 °C was defined as the point where the dielectric constant deviates from the Curie-Weiss equation (*γ* = 1), as shown in Fig. 2(b).

The birefringence Δ*n* induced by the QEO effect can be expressed by equation (3).


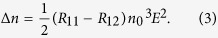


**Figure 3:**
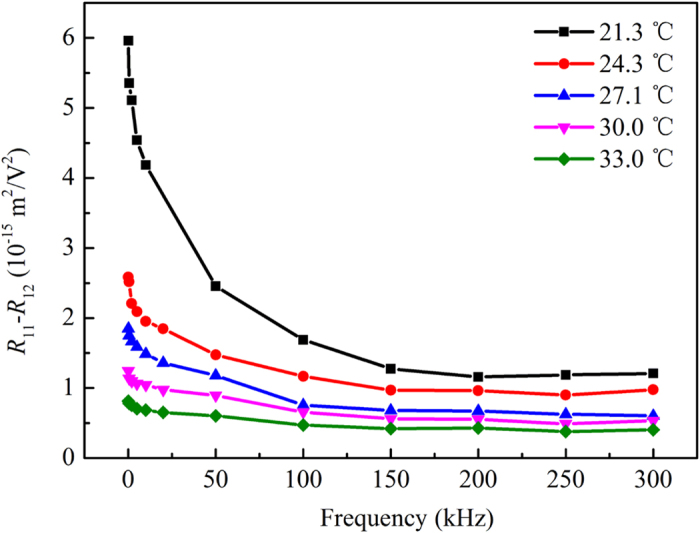
The frequency dependence (500 Hz–300 kHz) of the QEO coefficient *R*_11_ − *R*_12_ in a KTa_0.61_Nb_0.39_O_3_ crystal at the temperature range 0 °C < T − Tc < 13 °C.

**Figure 4:**
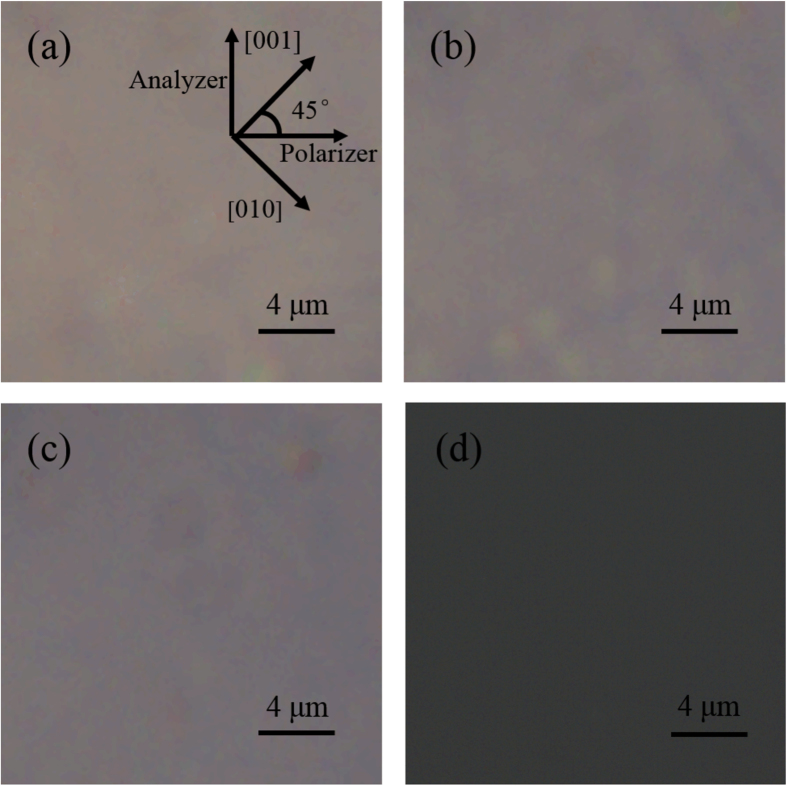
Polarizing microscope images of the crystal at temperatures (a) 21.3 °C, (b) 27.1 °C, (c) 33.0 °C and (d) 65.0 °C. The directions of the Polarizer and Analyzer were perpendicular to each other. The angle between directions of the crystallographic [001] (or [010]) and the Polarizer was π/4.

The contribution of PNRs to the QEO effect at the temperature range 0 °C < *T*-*T*_*c*_ < 13 °C was investigated using the frequency dependence (500 Hz−300 kHz) of *R*_11_ − *R*_12_, shown in Fig. 3. The coefficient *R*_11_ − *R*_12_ reached 5.96 × 10^−15^ m^2^/V^2^ at low frequency (500 Hz, 21.3 °C) and gradually decreased to a nearly stable value as the frequency increased to 300 kHz. Owing to the essentially local composition fluctuation of KTN, the size distribution of PNRs in the crystal varies near *T*_*c*_. As the frequency increases at a constant temperature, PNRs of relatively large size cannot respond to the electric field; consequently, the QEO coefficient decreases gradually to reach a stable value until none of the PNRs contribute to the QEO effect. The contribution of the PNRs to the QEO effect was estimated by calculating the attenuation factor *β*


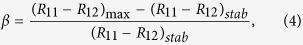


where (*R*_11_ − *R*_12_)_max_ is the maximum of *R*_11_ − *R*_12_ and (*R*_11_ − *R*_12_)_*stab*_ is the stable value of *R*_11_ − *R*_12_ at high frequencies. The attenuation factors at different temperatures were listed in Table 1. It was found that higher temperatures corresponded to lower attenuation factors; this was mainly because as the temperature increased, the average size of the PNRs decreased and fewer PNRs remained to contribute to the QEO effect. This was evidenced by the evolution trend of the PNRs with the temperature change, which was observed with a polarizing microscope and was shown in Fig. 4.

In the Fig. 4(a–d), the light transmitted through the Polarizer, crystal and Analyzer in sequence. As shown by the inset of Fig. 4(a), the directions of the Polarizer and Analyzer were perpendicular to each other. The angle between directions of the crystallographic [001] (or [010]) and the Polarizer was π/4. When the average size of the PNRs is far smaller than the wavelength, the PNRs densities along the six crystallographic directions ([001], [010], [100], [00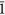], [0
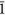
0] and [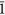
00]) could be regarded isotropic. The refractive indices along the [001] and [010] directions are equal and the polarization of the light would not be affected by the crystal. Thus the light would be extinct after the Analyzer. As the average size of PNRs increases, the isotropy of PNRs densities decreases. The difference between the refractive indices along the [001] and [010] directions increases gradually and the polarization of the light is affected by the crystal. Then the light would not be extinct totally. As observed in Fig. 4(a–d), with the increasing temperature, the darker and darker brightness of the image means that the extinction became more complete and the isotropy of PNRs densities increased gradually, i.e., the average size of PNRs decreased.

**Figure 5:**
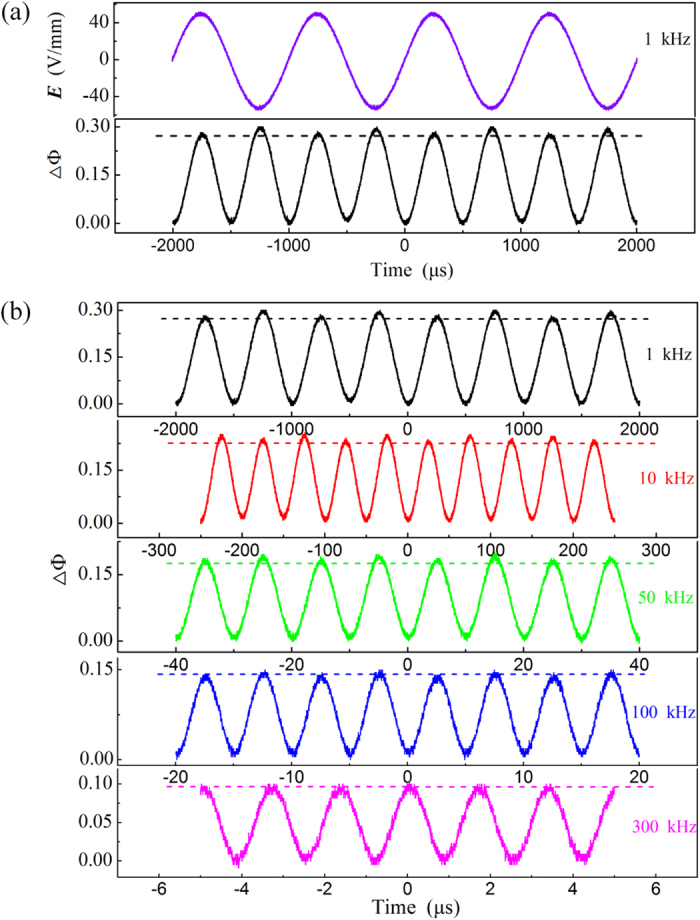
(**a**) The waveforms of the electric field (violet curve) and the induced phase change ΔΦ (black curve), as monitored by the oscilloscope under an AC electric field of 1 kHz. (**b**) The waveforms of ΔΦ under an AC electric field of 1 kHz, 10 kHz, 50 kHz, 100 kHz and 300 kHz. The horizontal dashed lines are marked to help observe the distortion phenomenon of QEO effect.

Furthermore, a distortion phenomenon of the QEO effect corresponding to the asymmetric distribution of PNRs was also observed at the low frequency of the electric field. Figure 5 shows the phase change ΔΦ induced by the QEO effect under electric fields of different frequencies at 21.3 °C.


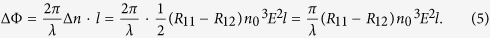


It is well known that the phase change induced by the QEO effect is independent of the direction of the electric field in the paraelectric phase. Therefore, the induced phase change ΔΦ should have the same peak value for both electric field maximum ±*E*_max_ along the position and negative directions. However, Fig. 5 shows that the peaks at ±*E*_max_ differ. This is attributed to an overlay of linear EO signals induced by the asymmetric distribution of PNRs along the electric field directions. This phenomenon is relatively obvious at low frequency and gradually disappears towards high frequencies. The distortion of the QEO effect disappears entirely when the frequency reaches 300 kHz, exactly the value where the QEO coefficient becomes completely stable. This observation supports that the distortion was indeed induced by the PNRs. More details about the distortion are discussed subsequently.

To better understand the mechanism of how the PNRs contribute to the QEO effect in KTN, a phenomenological model is proposed. The dipoles within each PNR combine and their polarization directions are coincident. According to G. Burns and F. H. Dacol^21^, the refractive indices along the *x* and *y* directions can be expressed by equation (6) in terms of the polarization intensity.


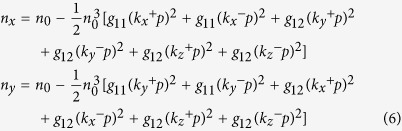


where *p* is the average moment of each PNR at a certain temperature, *k*_*x*_^+^, *k*_*x*_^−^, *k*_*y*_^+^, *k*_*y*_^−^, *k*_*z*_^+^ *and k*_*z*_^−^ are the densities of PNRs along the six directions of spontaneous polarization and *g*_11_ and *g*_12_ are the polarization-optic coefficients. The electric field is applied along the *x* direction. The polarization along the *y* and *z* directions is perpendicular to the electric field and responds equally to the positive and negative electric field; therefore, it does not contribute to the distortion of the QEO effect. For simplification, we consider only the asymmetric polarization distribution along the *x* direction, namely *k*_*x*_^+^ ≠ *k*_*x*_^−^ and *k*_*y*_^+^ = *k*_*y*_^-^  = *k*_*z*_^+^ = *k*_*z*_^−^ = *k*.

The polarization is redistributed under the electric field. The polarization perpendicular to the electric field is oriented along the direction of the electric field to reduce the potential energy, while the thermal kinetic energy tends to retain the initial disordered polarization distribution. When the system reaches equilibrium, the PNRs density *k*_⊥_ along the *y* and *z* directions can be expressed as:


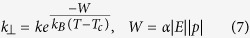


where *k*_*B*_ is the Boltzmann constant, *W* is the driving energy which is proportional to the product of the electric field *E* and the average moment *p*, *α* is the ratio constant. The moment *p* along the *x* direction would increase a variation when its direction is along the direction of the electric field or would decrease a variation when its direction is reverse to the direction of the electric field. Then, the refractive indices along the *x* and *y* directions become


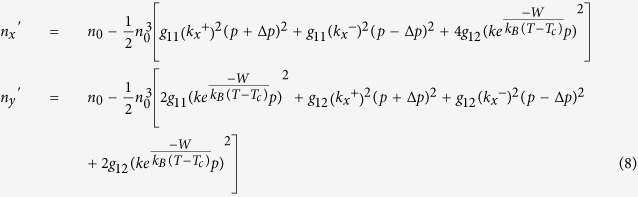


where Δ*p* = *ηE* is the moment variation along the *x* direction and *η* is the AC electric susceptibility of each PNR along the electric field direction. Then, the birefringence induced by the electric field can be expressed as


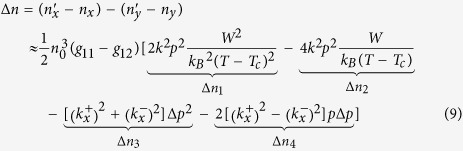


Here, 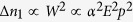, 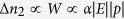, 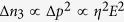 and 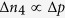 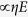. The terms Δ*n*_1_ and Δ*n*_3_ of the right side of equation (9) are proportional to the square of the electric field and contribute to the QEO effect although they are produced by the redistribution of the polarization perpendicularly to the electric field and by the polarization variation along the electric field, respectively. The term Δ*n*_2_ is proportional to the electric field but is independent of its direction. The term Δ*n*_4_ is also proportional to the electric field and is associated with the direction of the electric field. When the electric field reverses its direction, the term Δ*n*_4_ also changes its sign. So the birefringence induced by the electric field ±*E* is Δ*n*_1_ − Δ*n*_2_ − Δ*n*_1_ − |Δ*n*_4_| and Δ*n*_1_ − Δ*n*_2_ − Δ*n*_1_ + |Δ*n*_4_|, respectively. Thus, the phase changes ΔΦ induced by ±*E* would differ. It must be noted here that although both the Δ*n*_2_ a*n*d Δ*n*_4_ produce an overlap of linear EO signals on the QEO effect, the former would not change its sign as the electric field reverses, so only the latter can induce the distortion phenomenon described above. On the other hand, the linear EO effect induced by the Δ*n*_2_ has previously been observed by D. Pierangeli *et al*. in KTN, which is coincident with our model^1^. The frequency dependence of the QEO coefficient and the distortion phenomenon could also be easily interpreted with this model. As the frequency of the electric field increases, PNRs cannot respond to the electric field gradually, the constant *α* and *η* decrease to be nearly zero. Simultaneously, the QEO coefficient decreases to a stable value without contribution from the PNRs and the distortion phenomenon disappears.

## Conclusion

In conclusion, the dynamic response of PNRs in KTa_0.61_Nb_0.39_O_3_ crystals under an AC electric field was investigated in the frequency range 500 Hz−300 kHz near *T*_*c*_ (0 °C < *T* − *T*_*c*_ < 13 °C). The giant QEO coefficient was proved to be closely associated with the response of PNRs to the electric field. As the frequency increases, the PNRs cannot respond to the electric field and the QEO effect decreases gradually. With the temperature increasing, the contribution ratio of PNRs to the electro-optic effect also decreases. Besides, a distortion of the QEO effect was observed at low frequencies and disappeared gradually towards high frequencies. A phenomenological model is proposed to interpret the mechanism of how PNRs contribute to the QEO effect. This model is consistent with the observed results in this paper and also with those of D. Pierangeli *et al*.^1^ Further, this work could also contribute to interpret other interesting phenomena associated with PNRs near the phase transition boundary for other ferroelectric materials, in which PNRs have been reported, such as Ba(Zr_1−*x*_Ti_*x*_)O_3_, Pb(Zr_1−*x*_Ti_*x*_)O_3_ and (1 − *x*)Pb(Nb_2/3_Mg_1/3_)O_3_-*x*PbTiO_3_. Although the mechanisms of these phenomena are not clearly understood, it is obvious that the key point to interpret them is the dynamic response of the PNRs to the electric field, which is exactly the purpose of this paper.

## Additional Information

**How to cite this article**: Tian, H. *et al*. Dynamic response of polar nanoregions under an electric field in a paraelectric KTa_0.61_Nb_0.39_O_3_ single crystal near the para-ferroelectric phase boundary. *Sci. Rep*. **5**, 13751; doi: 10.1038/srep13751 (2015).

## Figures and Tables

**Table 1:** The attenuation factor *β* at different temperatures.

_*T*/°C_	_21.3_	_24.3_	_27.1_	_30.0_	_33.0_
*β*	_3.93_	_1.86_	_1.75_	_1.34_	_1.02_
